# Ovarian Brenner Tumor: A Report of Two Cases and Literature Review

**DOI:** 10.7759/cureus.46613

**Published:** 2023-10-07

**Authors:** Latifah A Alamer, Omar Y Almukhadhib, Khalid A Al Zahrani, Maysoon Adham, Rahaf A AlMousa

**Affiliations:** 1 Obstetrics and Gynecology, King Fahd Hospital of the University, Khobar, SAU; 2 Obstetrics and Gynecology, Imam Abdulrahman Bin Faisal University, Dammam, SAU; 3 Obstetrics and Gynecology, King Abdulaziz Medical City Riyadh, Riyadh, SAU

**Keywords:** ovarian neoplasm, epithelial tumors, transitional cell tumor, ovarian brenner, brenner

## Abstract

Brenner tumors are relatively uncommon surface epithelial tumors of the ovary, accounting for less than 2% of all ovarian tumors. They may be of benign, borderline, or malignant nature as classified by the World Health Organization. Definitive diagnosis is made by histopathological examination after surgical excision, as it does not have pathognomonic imaging features. Due to the rarity of these tumors, reporting these cases may be beneficial to develop diagnostic and treatment criteria. We herein report two cases of Brenner tumor and discuss the available literature.

Two cases of Brenner tumor were reported in addition to the literature review. Electronic search in different databases was used, accessing published full free-text articles in the English language, between January 2010 and December 2017, with the following MeSH terms: ovarian Brenner, Brenner, and ovary Brenner.

Nineteen articles were located, of which seven articles were selected because they were consistent with the aims of the review. Twelve articles were excluded as they did not meet the aim of the review. Data from the reviewed articles were used to finalize the conclusive recommendations.

Brenner tumors are rare ovarian tumors that are diagnosed by histopathological examination. Radiological investigation has a negligible role in the diagnosis, as Brenner tumors exhibit nonspecific features in imaging studies. To date, surgical excision remains the primary modality in diagnosing and treating Brenner tumors. The clinical characteristics of Brenner tumors require more research to be fully understood.

## Introduction

Brenner tumors are relatively uncommon surface epithelial tumors of the ovary, accounting for 1.4% to 2% of all ovarian tumors [1]. They may be of benign, borderline, or malignant nature as classified by the World Health Organization [2]. The majority of Brenner tumors are unilateral and benign, as only 2% to 5% are malignant [3]. They are most commonly discovered incidentally, as most of the patients are asymptomatic [3]. Surgical excision and pathological review are required for diagnosis, as no specific radiological features were demonstrated for this type of tumor [4]. In this paper, we report two cases of benign Brenner tumors and describe our histological, radiological, and surgical findings.

## Case presentation

Case 1

A 43-year-old woman presented to the emergency department complaining of lower abdominal pain, more on the left side, score 5 out of 10, with gradual onset, not radiating, with no relieving nor exacerbating factors, for one month. It was associated with nausea and increasing urinary pressure and frequency. In addition, she had delayed menses for three months. A detailed history was obtained and showed that she had a history of a left ovarian cyst six years ago, and she was offered a surgical intervention but she refused. She was unknown to have any chronic medical illnesses with an obstetrics history of six full-term normal vaginal deliveries and four miscarriages.

**Figure 1:**
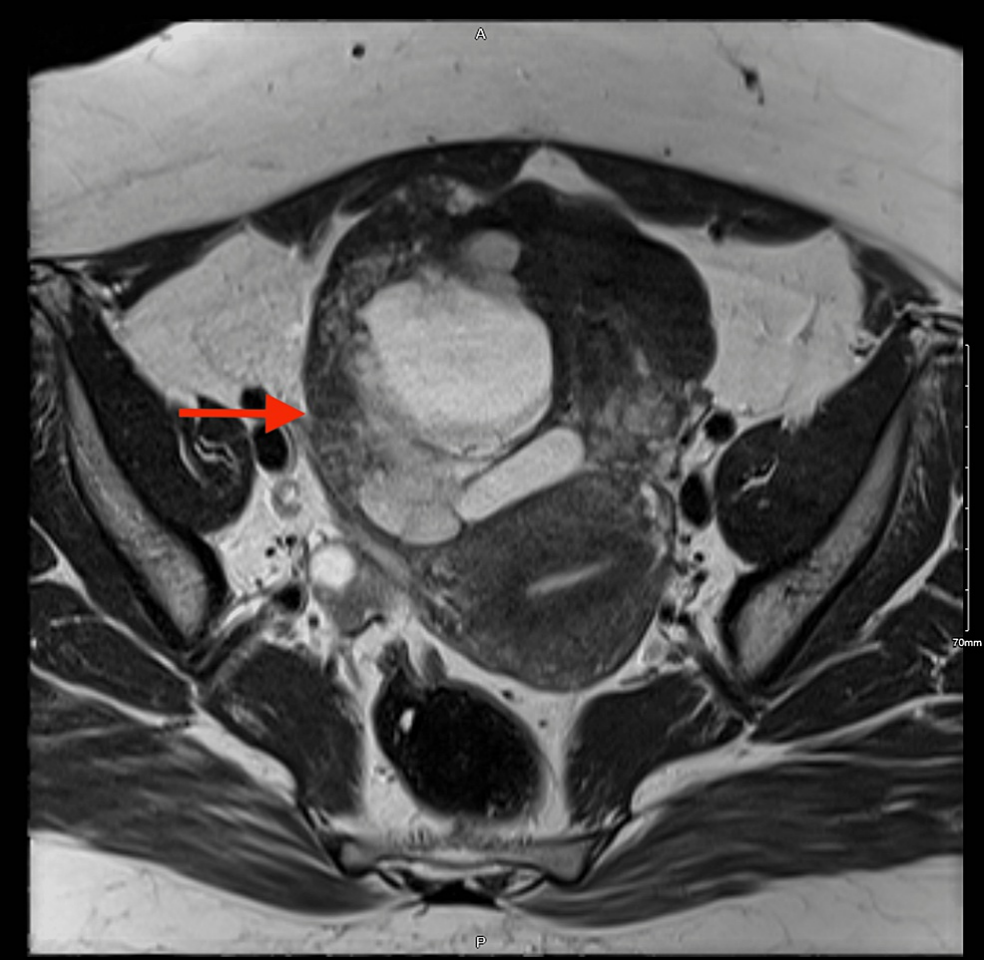
Magnetic resonance imaging (MRI) axial view. A large multiseptated cystic lesion is seen in the anterior central aspect of the pelvis located anterior to and abutting the uterus, capping and inseparable from the left ovary and extending anteriorly to the posterior aspect of the right rectus abdominis muscle. Laterally, the lesion is bounded on the left side by the stretched right round ligament; posteriorly, it is resting upon the urinary bladder dome and causing mild compression.

On physical examination, her vital signs were stable and she was not in pain or distress. The abdomen was soft and lax with no tenderness, and the gynecological examination was unremarkable. A pelvic ultrasound was performed, and a complex cystic mass was identified involving the left adnexa inseparable from the left ovary. The left adnexa was noted in the midline, and it measured approximately 8.9 cm by 6.3 cm by 8.4 cm. Pelvic magnetic resonance imaging (MRI) and computed tomography (CT) showed a large multi-septated cystic structure measuring 8.5 cm by 10.5 cm by 9.5 cm, mixed with peripheral endometriotic plaque seen in the anterior central aspect of the pelvis abutting the urinary bladder dome and anterior uterine wall inseparable from the left ovary, stretching the right round ligament and compressing the rectosigmoid junction (Figure 1). She was investigated in the line of ovarian tumors. Routine blood, tumor markers, and hormonal studies were normal. Tumor markers results were as follows: cancer antigen 125 (CA-125) at 15 U/mL (lab reference range: <35 U/mL), alpha-fetoprotein (AFP) at 2.4 ng/mL (lab reference range: <8 ng/mL), lactate dehydrogenase (LDH) at 142 U/L (lab reference range: 125-220 U/L), and beta-human chorionic gonadotropin (B-HCG) at <1.2 IU/L (lab reference range: 0-0.5 IU/L).

The patient underwent a laparotomy procedure. A large multilobulated mixed solid and cystic mass was noticed arising from the left ovary measuring approximately 11 cm by 9 cm by 10 cm. Left salpingo-oophorectomy was done and the specimen was sent for histopathological examination. Macroscopic examination of the specimen showed a tan-white multi-lobulated mass, weighing 254 g, and the cut surface showed firm fibrous multiple cystic areas filled with mucoid material. And microscopic examination revealed features of a benign Brenner ovarian tumor.

Case 2

**Figure 2:**
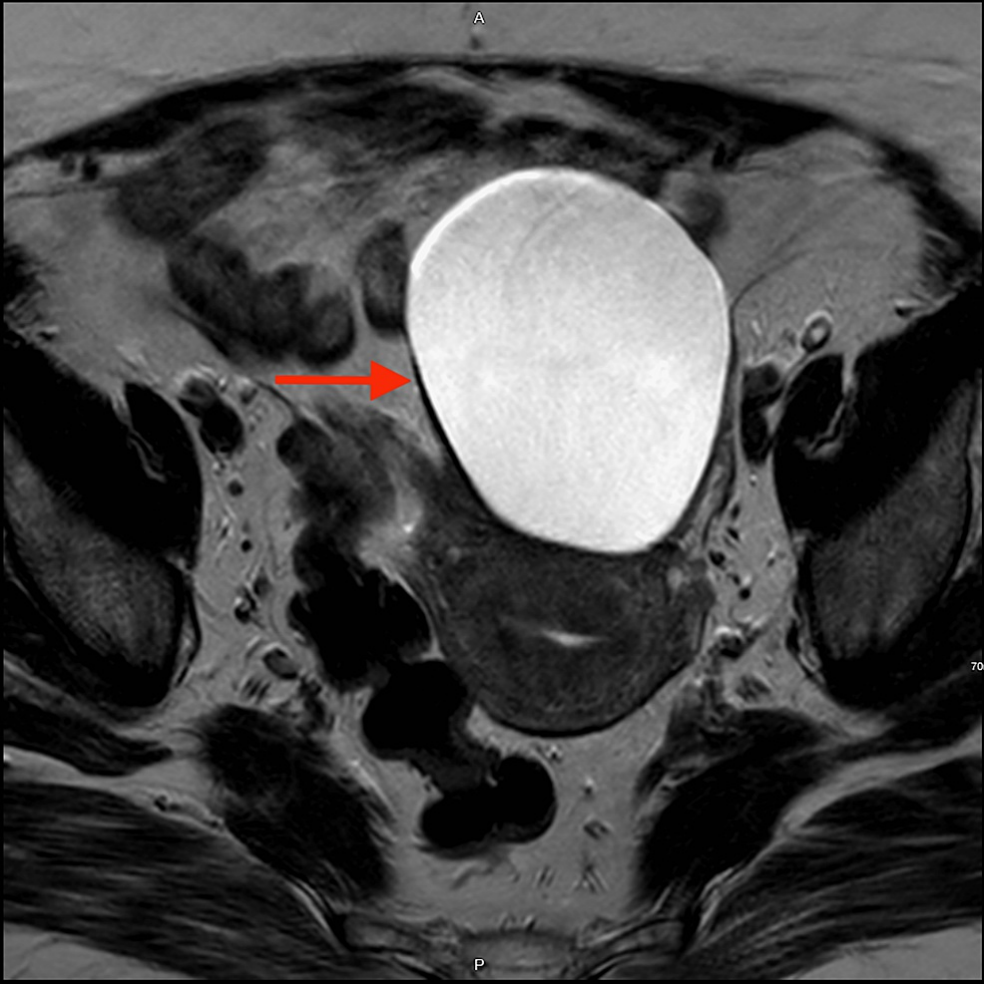
Magnetic resonance imaging (MRI) axial view. Left adnexal unilocular cyst mostly arising from the left ovary. It shows a thin-walled with no soft tissue component, papillary projection, or internal septations. T1 and T2 showed low and markedly high signal intensity, respectively, without diffusion restriction or enhancement.

A 62-year-old postmenopausal woman attended our hospital after she was diagnosed with a left ovarian mass on an ultrasound done in another hospital. She had been complaining of vague abdominal pain for the past three months, without other complaints or associated symptoms. She denied any weight loss or change in appetite. Her obstetric history included 12 full-term normal vaginal deliveries and one miscarriage. Her medical history included diabetes mellitus, hypertension, obesity (body mass index [BMI] 41), and, a surgical history of cholecystectomy. Upon examination, the abdomen was soft and lax, with no palpable masses. An official transabdominal and transvaginal ultrasound was performed, it showed a huge thin-walled cyst, seen in the midline more in the left adnexa, measuring 8.5 cm by 6.5 cm by 6.4 cm. After that, a pelvic MRI was done, which demonstrated an 8.6 cm by 6.5 cm by 6.1 cm unilocular thin-walled cyst with no soft tissue component, papillary projections, or internal septations mostly arising from the left ovary. There was no size significant lymph nodes or pelvic ascites (Figure 2). She underwent investigations for an ovarian tumor, and while tumor markers CEA, LDH, CA-125, and B-HCG were within normal ranges (CEA = 2.3 ng/ml, LDH = 204 U/L, CA-125 = 25 U/mL, B-HCG 3 IU/L), they were all considered negative for abnormalities, given their values fell within the respective reference ranges (CEA reference range <5 ng/mL, LDH reference range 125-220 U/L, CA-125 reference range <35 U/mL, and B-HCG reference range 0-0.5 IU/L). 

Given the aforementioned findings with no worrisome features of the cyst and considering the patient’s age, she was offered a total abdominal hysterectomy with bilateral salpingo-oophorectomy. Intraoperatively, there was a left ovarian cyst measuring 6 cm by 8 cm with a smooth surface. Histopathological examination revealed the following: inactive endometrium, uterine adenomyosis, left ovarian Brenner tumor, and a separate serous cystadenoma with no evidence of malignancy. She had an uneventful postoperative period and was discharged with regular follow-up.

## Discussion

For the first time, in 1907, Fritz Brenner described Brenner tumors in detail [5]. Brenner tumors are rare epithelial ovarian tumors, and between 1.4% and 2% of all ovarian tumors are thought to be Brenner tumors [6,7]. Brenner tumors can be found in women of any age, but mostly in the fifth to seventh decade of life and postmenopausal women [6-8]. The majority of Brenner tumors are discovered incidentally, as most of the patients are asymptomatic [1,2]. However, symptomatic patients may present with pelvic pain, mass, or abnormal uterine bleeding [8,9]. Brenner tumors originate from the surface epithelium of the ovary, which has the properties of the celomic epithelium of the ovary, which, in turn, is capable of undergoing metaplastic changes, forming typical urothelial-like epithelial elements of the neoplasm [10]. Association of a second ovarian tumor, for example, mucinous and serious tumors, with a Brenner tumor has been reported [11,12]. It has been classified into three subtypes: benign, borderline, or atypical proliferative Brenner tumor and malignant by the World Health Organization [13]. Nearly all of the Brenner tumors are benign, as borderline and malignant are uncommon [14]. According to the literature, approximately 1% of Brenner tumors are malignant [8].

Macroscopically, most benign Brenner tumors are unilateral and measure <2 cm in diameter. They exhibit a well-circumscribed, solid structure with a firm rubbery consistency; calcification and small cysts may be present [2]. Brenner tumor could be misdiagnosed as a dermoid cyst, for the reason that a dermoid cyst may contain calcifications [9]. They are typically composed of oval nests of epithelial transitional cells with fibromatous stroma. The nuclei are oval, and longitudinal distinct grooves are usually present, which is called coffee bean nuclei [12-14]. Moreover, it has been noticed that nuclei occur in various distinct forms with higher mitotic activity in malignant types of Brenner tumors [8]. In a small number of cases, a mutation in the KRAS gene at codon 12 was identified [15,16]. In addition, there have also been reports of associations between Brenner tumors and serous or mucinous cystadenomas [17].

**Figure 3:**
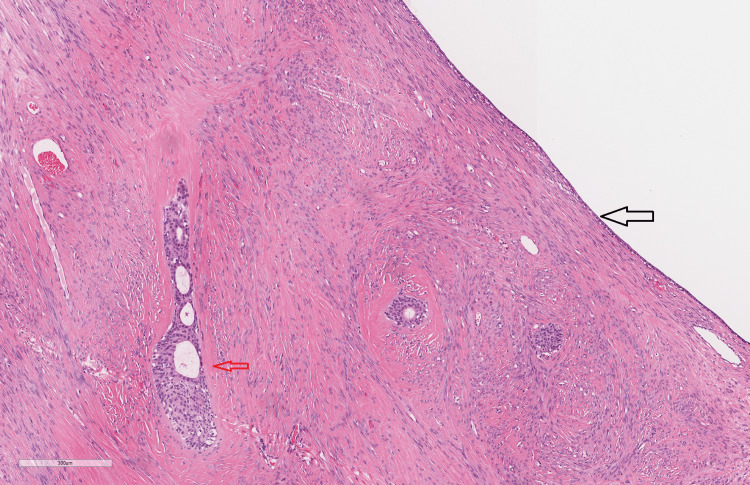
Case 1 histological image. Hematoxylin and eosin-stained section (x50 magnification) depicting small nests of transitional cells embedded in the fibrous stroma (red arrow), adjacent to a unilocular cyst lined by low cuboidal cells (black arrow).

**Figure 4:**
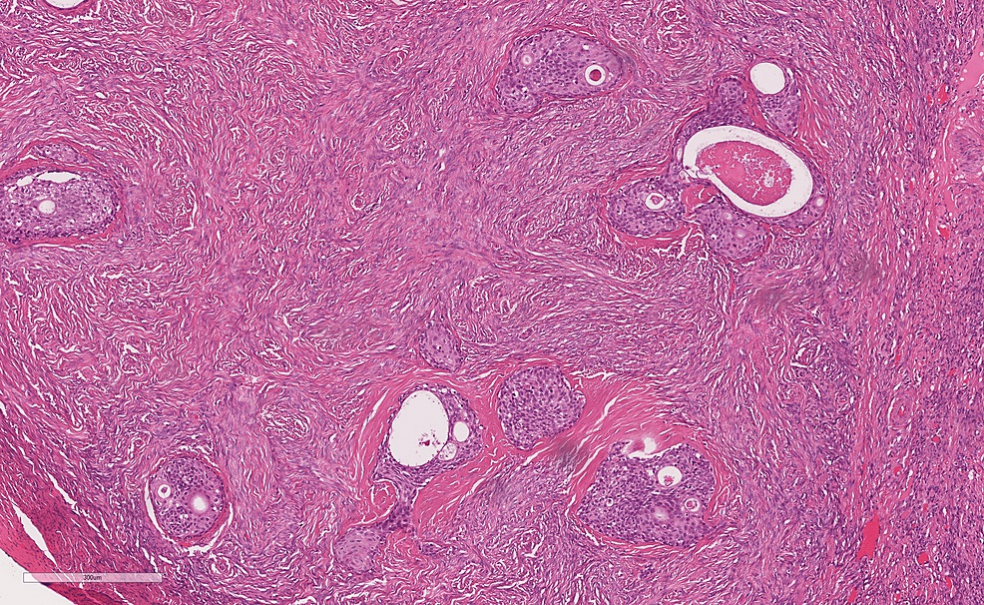
Case 2 histological image of a Brenner tumor. Hematoxylin and eosin-stained section (x100 magnification) showcasing a Brenner tumor.

**Figure 5:**
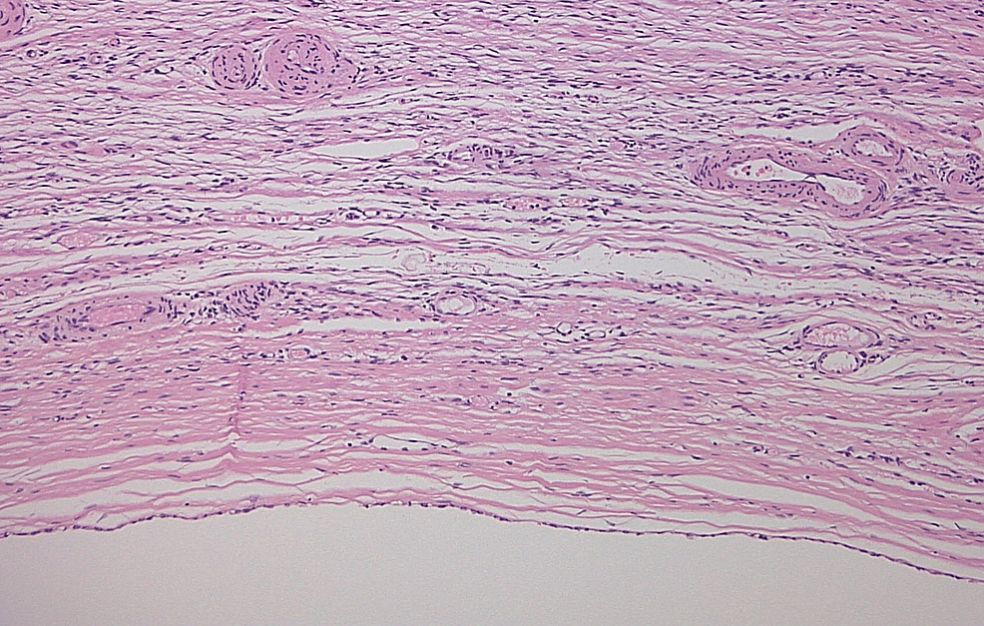
Case 2 histological image of a serous cystadenoma. Hematoxylin and eosin-stained section (x100 magnification) showcasing a serous cystadenoma with flattened epithelium.

Microscopically, the ovary in both cases exhibits two lesions, one of which displays the characteristic features of a Brenner tumor: compact, well-demarcated nests of monomorphic bland cells with round to ovoid nuclei reminiscent of urothelial cells. These nests are embedded in a fibrous stroma with occasional microcystic spaces containing mucinous material (Figures 3-4). The second lesion is a unilocular cyst lined by a simple epithelial layer ranging from low cuboidal to flattened cells, consistent with a serous cystadenoma (Figures 3-5). However, the distinction between the two cases is that the first case comprises a Brenner tumor that intermingles with the component of a serous cystadenoma, as illustrated in Figure 3. On the other hand, in the second case, two distinct lesions are present within the same ovary. One of these displays a classic Brenner tumor (Figure 4), while a separate serous cystadenoma component is seen as well (Figure 5).

Borderline Brenner tumors are larger than benign tumors, ranging from 10 to 28 cm. Typically, they are cystic tumors with some solid areas with significant atypia and mitotic activity [14]. Only 4% to 5% of Brenner tumors are borderline; this represents only a small fraction of the total tumor population. It is usually confined to a single ovary. It has been found that borderline and malignant Brenner tumors are more common in older women compared to benign Brenner tumors. The majority of borderline Brenner tumors are detected in the first stage, and surgical treatment of borderline Brenner tumors usually leads to a favorable outcome [16].

Malignant Brenner tumors are usually of transitional cell type, resembling an invasive urothelial carcinoma, associated with a benign or borderline Brenner tumor component [18]. At the time of diagnosis, about 80% are confined to the ovary (stage I), and 12% are bilateral. Hormonal activity has been reported with Brenner tumors, and estrogen is considered the main hormone secreted by these tumors [7]. This, in turn, may lead to endometrial pathology as endometrial hyperplasia, which was reported in 4% to 14% of the cases [19]. Moreover, androgen production is extremely rare, but it has been reported [7]. CA-125 tumor marker was a useful tool for detecting these tumors [6]. Malignant Brenner tumors are rare, so adjuvant therapy for these tumors is unclear since clinical encounters with them are rare and no standard treatment has been developed [13]. In comparison to other epithelial ovarian tumors, Brenner tumors have a better prognosis [8].

It is difficult to diagnose Brenner tumors by radiological studies, as there is no specific pathognomonic feature for these tumors [8]. Imaging tests typically reveal that benign Brenner tumors resemble other solid ovarian masses such as fibromas, fibrothecomas, and pedunculated leiomyomas [17]. Furthermore, imaging characteristics between benign and malignant Brenner tumors have been inadequately described [13]. Surgical excision is diagnostic as well as curative for Brenner tumors, with surgical staging required for malignant tumors [18].

## Conclusions

Benign Brenner tumors are rare ovarian tumors that are diagnosed by histopathological examination after surgical excision. Radiological investigation has a negligible role in the diagnosis as Brenner tumors have nonspecific features in imagining studies. We are reporting two cases of Brenner tumors, aiming to contribute additional information to the existing knowledge on this topic.
